# Thermal Emission Control via Bandgap Engineering in Aperiodically Designed Nanophotonic Devices

**DOI:** 10.3390/nano5020814

**Published:** 2015-05-20

**Authors:** Enrique Maciá

**Affiliations:** Materials Physics Department, Faculty of Physics, University Complutense of Madrid, E-28040 Madrid, Spain; E-Mail: emaciaba@fis.ucm.es; Tel.: +34-91-394-4745; Fax: +34-91-394-4547

**Keywords:** thermal emission, aperiodic order, nanophotonics

## Abstract

Aperiodic photonic crystals can open up novel routes for more efficient photon management due to increased degrees of freedom in their design along with the unique properties brought about by the long-range aperiodic order as compared to their periodic counterparts. In this work we first describe the fundamental notions underlying the idea of thermal emission/absorption control on the basis of the systematic use of aperiodic multilayer designs in photonic quasicrystals. Then, we illustrate the potential applications of this approach in order to enhance the performance of daytime radiative coolers and solar thermoelectric energy generators.

## 1. Introduction

During the last few years, the search for materials exhibiting quite specific thermal emission properties has been spurred on by the necessity of attaining high-performance devices for light harvesting and thermal energy control. Thus, devices displaying either very low or very high emissivity values over relatively broad frequency windows are usually required. For instance, efficient daytime radiative coolers must radiate heat to outer space through a transparency window in the Earth’s atmosphere ranging between 8 and 13 μm, but they should also reflect most incoming solar radiation within the UV and visible wavelengths [1]. Unfortunately, no known existing natural materials are able to perform this way, so one must synthesize them on purpose, and the original search turns into a materials engineering task.

A very convenient way to address this task consists in coating a suitable bulk material with a properly designed multilayered thin film, in order to exploit the beneficial optical properties contributed by each component in a synergetic way. Within this approach the coat can act either as a passive filter (when the refractive indices of the layers material are all real valued) or as an active emitter (when at least one of the layers material in the film has a complex valued refraction index). In both cases, the resulting thermal radiation spectrum of the composite structure can be substantially modified as compared to that corresponding to the original substrate alone, due to resonance effects within the multilayered coat [2].

These appealing results were originally obtained by considering a periodic sequence of layers in the thin film coat. Though the recourse to periodic order is quite convenient for the sake of simplicity, during the last decade it has been progressively realized that ordered structures can be suitably expanded to embrace not only periodic arrangements but aperiodic ones as well. In this way, the very notion of aperiodic order, that is, order without periodicity, has been explored to properly describe a growing number of physical systems, including metallic and dielectric multilayers [3,4,5,6,7,8]. Thus, well-known materials still remain in use, along with the previously gained technological expertise, as the attention is entirely focused on the (relatively low-cost) aspects related to device architecture optimization.

A typical example of an ordered aperiodic multilayer is provided by a structure consisting of a number of layers stacked according to a certain deterministic rule, for instance, one given by a substitution sequence. A simple example of such a nanostructured material is a two-component Fibonacci multilayer, where layers of two different materials (metallic, semiconductor, dielectric, ferroelectric) are arranged according to the celebrated Fibonacci substitution rule A→AB and B→A, whose successive application generates the sequence of layers A → AB → ABA → ABAAB → ABAABABA → ABAABABAABAAB → ... and so on. The number of layers in a sequence of order *j* is given by the Fibonacci number *F*_*j*_, which is obtained from the recurrence relation *F*_*j*_ = *F*_*j*−1_ + *F*_*j*−2_, *j* ≥ 2, with *F*_0_ = *F*_1_ ≡ 1. Another example of aperiodic multilayer is provided by the substitution rule A → ABA and B → BBB, generating the fractal triadic Cantor sequence.

A key feature of these man-made materials is the presence of two kinds of order in the same sample at different length scales: at the atomic level we have the usual crystalline order determined by the periodic arrangement of atoms in each layer, whereas at longer scales we have the aperiodic order determined by the sequential deposition of the different layers. This aperiodic order is imposed during the growth process and can be precisely controlled. Since different physical phenomena have their own relevant physical scales, by properly matching the characteristic length scales we can efficiently exploit the aperiodic order we have introduced in the system, hence opening new avenues for technological innovation.

In particular, due to their characteristic highly fragmented frequency spectrum, aperiodic multilayers provide more full transmission peaks (alternatively, absorption bands) than periodic ones in a given frequency range for a given system length. This feature stems from the richer structural complexity of aperiodic sequences (related to the presence of quasiperiodic and/or self-similar order related fingerprints), naturally leading to the presence of more resonant frequencies stemming from multiple interference effects throughout the structure. The existence of more frequencies for operation becomes particularly useful when looking for selective enhancement (suppression) of thermal emission for some selected frequencies. Thus, by properly exploiting the *additional degrees of freedom* inherent to the aperiodic arrangements, one can design optical devices offering a broader and more flexible *design capability* than their periodic counterparts for certain specific applications [3,4,5,6,7].

## 2. Aperiodicity by Design

In the early years, most studied aperiodic multilayers were based on the systematic application of certain mathematically inspired substitution sequences in order to ascertain their fundamental properties. As the field of aperiodic order based photonics is coming to maturity a logical extension naturally appears: it consists in considering structures whose layers are intentionally arranged according to purposely defined aperiodic designs, able to tailor their physical properties to fit some specified requirement. In so doing, convenient aperiodic orderings are created by hand, generally following suitable optimization algorithms, thereby substantially enriching the variety of aperiodic orders of interest in both fundamental and applied science domains. We will refer to the systems obtained in this way as aperiodic systems by design [3,5].

This procedure is an example of what is generally known as a reverse engineering problem. In such cases, the ideal structures are found by numerically solving an optimization problem where a number of optimization parameters (e.g., the layer thicknesses or the incidence angle) are treated as independent variables under certain physical constraints (e.g., the requirement of a small enough total length value for the multilayer). Then, a merit function to be optimized is defined in terms of the physical magnitudes of interest in the considered device. For instance, if one is interested in optimizing the reflectance *R*(λ) of a coating over a given spectral range one may use a function of the form:

(1) $$f=\frac{1}{N}\sum_{\ell =1}^{N}\left[R_{0}-R\left(\lambda_{\ell }\right)\right]{\;}^{2}$$

where *N* is the total number of layers, *R*_0_ is a reflectivity reference value, and λ_ℓ_ is the radiation wavelength propagating through the ℓ-th layer. Following this approach a number of Mo/Si aperiodic multilayers were designed and grown in order to attain a maximum reflectivity over a specified range of wavelengths or angles of incidence for their use as extreme-ultraviolet (XUV) radiation mirrors [9], or analyzers [10]. Similar approaches have been used to design efficient aperiodic W/Si multilayer mirrors for X-ray plasma diagnostic [11], or narrowband optical filters able to simultaneously tune multiple wavelengths [12]. More elaborated procedures, based on the application of the so-called genetic algorithm [13], have been successfully applied in order to design omnidirectional reflectors [14].

**Figure 1:**
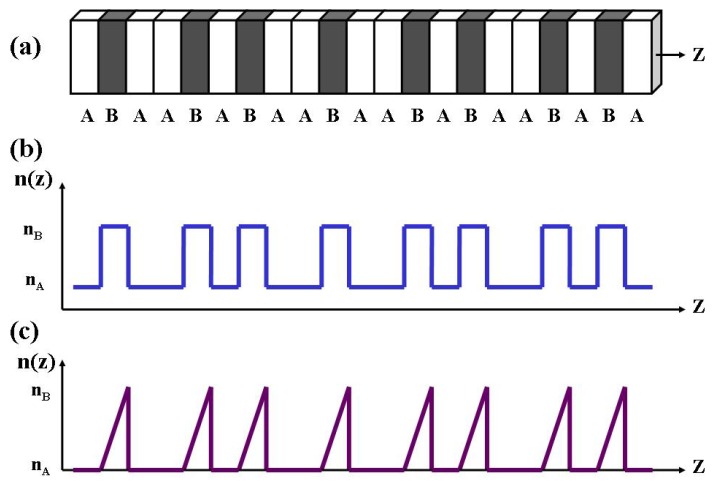
(**a**) Sketch of a Fibonacci dielectric multilayer grown along the *z* direction; (**b**) Refractive index profile *n*(*z*) for an arrangement of layers with constant refraction index; (**c**) Refractive index profile *n*(*z*) for an arrangement of layers with a linearly graded refraction index.

When trying to optimize some specific application by means of aperiodic order engineering a key question concerns the relationship between the structural order of the multilayer itself and its related physical properties. In multilayered optical devices the structural order naturally translates in terms of the refractive index spatial profile *n*(*z*) function, where *z* is the stacking direction (Figure 1). Whereas aperiodic substitution sequences can only define relatively restricted types of aperiodic refractive index profiles, the recourse to analytical functions including suitable free parameters allows for a more general treatment, so that one can describe different kinds of refractive index distributions within a unified mathematical approach.

As an illustrative example one can consider a binary multilayer composed of layers whose refraction index values are assigned depending on the sign of the auxiliary function *V*_ℓ_ = cos(απℓ^ν^) where ℓ labels the layer sequence, α and ν being real parameters, so that *n*_ℓ_ → *n*_A_ if *V*_ℓ_ ≤ 0, and *n*_ℓ_ → *n*_B_ otherwise [15]. For ν = 1 and rational α one has a periodic refractive index profile, whereas for an irrational α value the multilayer sequence becomes quasiperiodic. In addition, for ν < 1 (alternatively, ν > 1) the refractive index profile is described by an aperiodic modulation whose wavelength progressively increases (decreases) as ℓ increases. Accordingly, by properly adjusting the values of the design parameters α and ν we can obtain a great diversity of aperiodic refractive index profiles. Thus, the parameter ν can be regarded as an exponent controlling the aperiodicity degree of the considered structure. Numerically obtained transmittance curves as a function of ν have been recently reported for multilayers based on this sequence in a systematic way (with α = $\frac{\sqrt{5}-1}{2}$, the reciprocal of the golden mean). The obtained results show the existence of a characteristic ν value leading to averaged reflectivity values larger than those corresponding to both periodic and random structures of comparable size [16].

## 3. Thermal Emission Control

**Figure 2:**
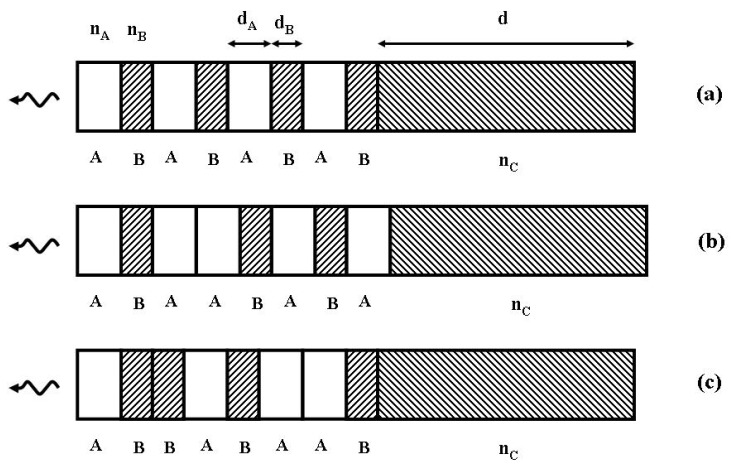
A multilayered stack (thin film) made of alternating layers of refractive indices *n*_A_ (white layers) and *n*_B_ (shadowed layers) coats a thick absorbing substrate of refraction index *n*_C_ (complex). The entire structure is embedded in a medium, taken to be air (*n* = 1). Different working regimes are obtained depending on the adopted layers distribution. In (**a**) the layers are periodically distributed, whereas in (**b**) and (**c**) they are respectively arranged according to the Fibonacci and Thue-Morse (A **→** AB, B **→** BA) sequences.

As it is well-known, even in the case of highly absorptive compounds, not all incident radiation is usually absorbed by a given material. This is because the material reflects some radiation at its surface. Therefore, most absorbing materials exhibit emissivity values lower than unity. An ingenious way to circumvent this shortcoming, which allows one to enhance the thermal emissivity of a given material at certain frequencies, was proposed on the basis of the structural design depicted in Figure 2. As it was previously discussed, the main idea is to coat the bulk substrate with a thin film made of alternating layers of different materials. In so doing, one aims to exploit quantum confinement effects in order to modify the emission processes through the multilayered structure, thereby properly engineering the thermal radiation characteristics of the overall system.

**Figure 3:**
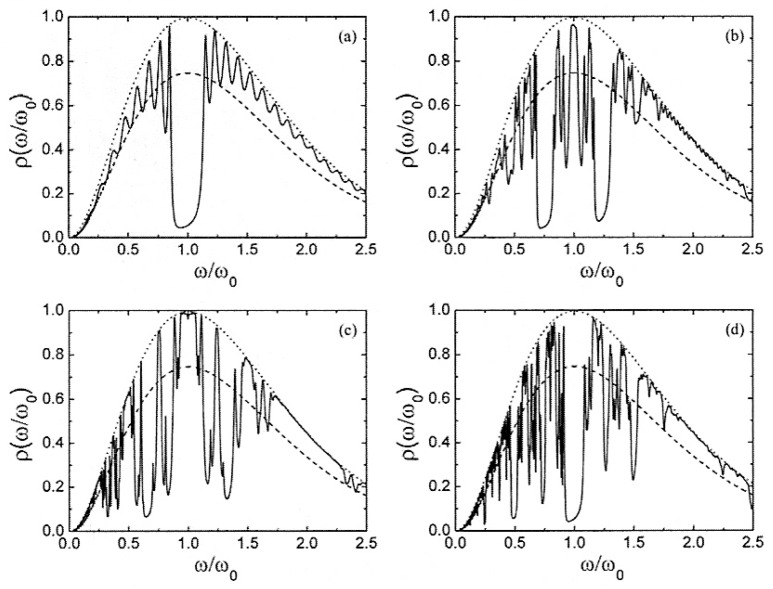
Thermal radiation spectra as a function of the reduced frequency under normal incidence conditions for the devices shown in Figure 2 with *n*_A_ = 1.45 (SiO_2_) and *n*_B_ = 1.0 + 0.01*i*, where the thin film coat layers are respectively arranged according to the following sequences: (**a**) Periodic; (**b**) Fibonacci (*N* = 377). (**c**) Thue-Morse (*N* = 512); and (**d**) period-doubling (*N* = 512). The perfect blackbody thermal spectrum is given by the dotted curve, whereas the dashed curve gives the thermal spectrum of the substrate, with refractive index *n*_C_ = 3 + 0.03*i*. The temperature is chosen so that the blackbody (Wien) peak is aligned with the midgap frequency ω_0_ = 2π*c*/λ_0_ (λ_0_ = 700 nm). All the curves are properly normalized by this peak power. (Adapted from [17]. Copyright Courtesy of Eudenilson L. Albuquerque, 2007).

A convenient way to deal with this system within the framework of the transfer matrix technique relies on the evaluation of the so-called thermal optical power:

(2) E(ω)=1−R(ω)−T(ω)

where *R*(ω) and *T*(ω) are the reflectance and transmittance, respectively. *E*(ω) is physically interpreted as the ratio of the optical power emitted at frequency ω into a spherical angle element dΩ by a unit surface area of the thin film, to the power emitted by a blackbody with the same area at the same temperature. In this way, the power spectrum of the multilayered film located in front of an emitting hot surface (Figure 2) is given by the Equation (2):

(3) $$P(\omega ,T)=E(\omega )P_{B}(\omega ,T)$$

where

(4) $$P_{B}\left(\omega ,T\right)=\frac{\hbar \omega^{3}}{2\pi^{2}c^{2}}\frac{1}{e^{\frac{\hbar \omega }{k_{B}T}}-1}$$

is the Planck’s law [2]. Some illustrative results are shown in Figure 3.

Let us first consider the case where a periodic film coating sits atop the heated substrate (Figure 3a). As we see the film significantly blocks heat radiation emitted by the substrate at the frequencies corresponding to the photonic crystal bandgap (ω/ω_0_ = 1) as expected, but we also observe that the substrate’s emission is enhanced from the gray-body level all the way up to the perfect blackbody rate at a number of frequencies corresponding to the pass-band transmission resonances of the multilayered film. This occurs because the thin film acts as an antireflective coating at these resonances. In this way, all the incident radiation tunnels through the multilayer structure into the substrate for these selected frequencies, so that the substrate effectively behaves as a perfectly absorbing blackbody in that case [2,17] A similar enhancement of the substrate's thermal emittance at certain resonance frequencies, accompanied by the corresponding inhibition at the stop-bands, is observed in the aperiodically arranged thin film coatings as well. In the case of the Fibonacci coating (Figure 3b) a characteristic trifurcation splitting can be clearly appreciated around a number of frequencies, and one finds a strong emittance within the spectral range corresponding to the midgap in the periodic case. In fact, the presence of allowed bands in certain forbidden regions of the periodic system is a characteristic feature of quasiperiodic systems [3,4,5,6,7]. An analogous pattern is observed in the thermal spectrum corresponding to the Thue-Morse thin film, whereas that corresponding to the period-doubling sequence is more similar to the periodic one around the ω/ω_0_ = 1 spectral range. In all the aperiodic arrangements, however, one has a richer thermal emission spectrum, due to the highly fragmented nature of their transmission profiles. Quite interestingly, these spiky thermal emission spectra can be substantially smoothed (hence obtaining broader spectral ranges with enhanced emittance) by using metamaterials in the composition of aperiodic multilayer coats [17,18].

## 4. Possible Applications

### 4.1. Daytime Radiative Coolers

A passive cooler is a device which decreases its temperature below that of the ambient air without any electricity (or any other form of energy) input. Passive cooling was first demonstrated at night using a technique known as radiative cooling, in which a device exposed to the sky is used to radiate heat to outer space through a transparency window in the atmosphere ranging between 8 and 13 μm [19]. Daytime radiative cooling under direct sunlight was subsequently shown by properly extending the same fundamental technique using a photonic radiative cooler. To this end, a thin film multilayer composed of seven alternating layers of hafnium dioxide (high refraction index) and silicon dioxide (low refraction index) of varying thicknesses were deposited on top of a 200 nm silver substrate. The resulting coating was in turn deposited on top of a 750 μm thick, 200 mm diameter silicon wafer. The bottom four layers in the coating film have thicknesses within the range 10–70 nm, and assist in optimizing solar reflection in a manner akin to that used in periodic one-dimensional photonic crystals. The top three layers are one order of magnitude thicker (within the range 200–700 nm) and are primarily responsible for thermal radiation from the cooler device. Thus, it is the presence of two different kinds of layer sizes which ultimately accomplishes the two functional tasks required for the coating, namely, high solar reflectance (due to the shorter layers stack) and high infra-red emissivity within the 8–13 μm window (due to the longer layers stack). Both the total number and the proper thickness values of the alternating low/high refraction index layers was determined by using a numerical optimization method. Making use of this device a cooling of about 5 °C below ambient air temperature, amounting to 40 Wm^−2^ cooling power, has been reported under direct sunlight [1,20].

These promising figures were obtained by properly combining suitable material properties and interference effects in an integrated structure that collectively achieves high solar reflectance (97% at normal incidence) and strong thermal emission in the selected infra-red frequency window. Nevertheless, some room is still left for further improvement (say, full solar reflectance along with perfect transmission through the Earth’s atmosphere infra-red windows), and one may wonder if these goals may be achieved by considering alternative layer sequence *orderings* for the different layers. Indeed, the possibility of attaining a broadband reflectance, covering a nearly full-visible spectrum window has been recently demonstrated in a TiO_2_ nanotube-based photonic crystal whose lattice constant is *gradually* decreased according to an *arithmetic series* of the form *d*_ℓ_ = *d*_0_ + (ℓ − 1)*d*, ℓ = 1, …, *N*, where *d*_0_ is the thickness of the first layer and *d* is the common difference. In this way, a flattened reflectance peak reaching 100% intensity within the range 400–600 nm was numerically obtained by properly ensambling two different slabs containing *N* = 8 layers (with *d* = 3.6 nm, and *d*_0_ = 165.2 nm or *d*´_0_ = 194 nm, respectively) to form two *N* = 16 bilayers, which were in turn stacked to each other to get a *N* = 32 multilayer with a total length *L* = 819.2 nm [21]. Note that though the total length of this graded multilayer reflector amounts to about four times that corresponding to the solar reflector component used in the daytime cooler device in Reference [20] (*i.e.*, *L* = 174 nm), its characteristic *modular design* can readily explain its ability to ultimately outperform the latter one. In fact, this property can be readily understood by regarding the overall structure as a modular stack of shorter photonic crystals with different lattice parameters, each one reflecting light with a peak located at its respective Bragg resonance condition. Thus, by properly selecting the location and width of each module main reflectance peaks, one may design a much broader, almost flat, reflectance spectrum profile, naturally stemming from the different modules spectra superimposition.

Further elaborating on this physical picture it was numerically shown that an even wider full reflectance band (ranging from 380 to 780 nm) could be obtained by considering hierarchical photonic crystals consisting in geometrically distributed layers, where the aperiodic order of the *n*(*z*) profile is given in terms of a *geometric serie*s rather than an arithmetic one [22]. In this study the influence of the angle of incidence and the light polarization was carefully analyzed, and the refraction index dispersion effects in the layers was explicitly taken into account, hence providing a quite realistic description. Geometric series naturally introduce a scale factor in the resulting refraction index profile, a feature they share with fractal structures characterized by self-similar symmetry. Accordingly, it is reasonable to expect that additional improvement in both the solar reflectance and the infra-red thermal emission properties could be attained in radiative cooling devices able to exploit the additional design degree of freedom provided by the scalability property characteristic of fractal multilayers geometries [23,24]. Thus, it has been shown that, for every type of fractal structure, the transmission spectrum of any given generation *k* > 2 contains embedded transmission spectra of all preceding generations *j* = 1, 2, …, *k* − 1, the spectrum of every preceding generation being squeezed along the frequency axis by a scale factor *s*^*k*−*j*^ [25,26,27,28]. The progressive fragmentation of the frequency spectrum as k increases gives rise to narrower transmission bands, which in turn result from preferential localization of light-wave energy in certain resonant cavities present in the structure. In fact, one easily realizes that the triadic Cantor second generation structure can be regarded as a BBB cavity embedded by a pair of ABA mirrors. For higher generations the number of existing cavities grows by a factor of three and the related transmission peaks split accordingly.

Finally, an additional step in this route towards higher order designs, one may consider quasiperiodic structures, which combine the self-similar features typical of fractal geometries with the very possibility of including graded refraction index profiles. A suitable example is illustrated in Figure 1c and it consists in two kinds of layers: one has a constant refraction index (*n*_A_) and the other has a linearly varying refraction index given by the expression,

(5) $$n_{B}=n_{A}+\frac{n_{B}-n_{A}}{d_{B}}z$$

where *d*_B_ is the B layer thickness [29]. Of course, instead of simply linear one may consider an exponential or any other refraction index gradation laws as well [30]. In turn, layers A and B are arranged according to a sequence prescribed by a given substitution rule (*i.e.*, Fibonacci, Thue-Morse, Period-doubling). In accordance with the obtained numerical results significantly broadband full reflectance profiles, covering a given spectral range, could be obtained by properly combining these aperiodic photonic crystals in modular designs.

### 4.2. Solar Cells and Solar Thermoelectric Generators

Thin film solar cells with thicknesses ranging from hundreds of nm to a few μm are attractive candidates for low-cost replacement of significantly thicker wafer-based devices, due to reduced costs of raw materials and processing. Light trapping photon management strategies rendering this cell optically thick enough despite its limited physical thickness is then crucial to attain high efficiency solar cells. To this end, the design of back-reflectors acting as diffraction gratings able to turn the incident light by an angle of 90° at specific frequencies (resonant photonic modes) that can be guided and thus absorbed along the plane of the cell have proved very useful devices. Though earlier designs were based in periodic layer sequences, more recent works have analyzed in detail the richness of quasiperiodic geometries, demonstrating that a nano-grating grown according to the Fibonacci sequence used as back-reflector in a thin film solar cell guarantees higher absorption enhancement with respect to the periodic counterpart over the whole incidence angle range, providing a slightly better performance of about 5% for angles less than 20° and for angles greater than 45° [31]. This improvement stems from the denser Bragg spectrum of Fibonacci lattices which allows to reach the phase match condition over more wavelengths, hence leading to a higher multiple resonance pattern for a given spectral range. This property also renders the quasiperiodic back-reflector more robust with respect to the incoming light incidence angle variations.

The aperiodic order based design strategy, used for metallic back reflectors to improve light absorption in thin film solar cells, can be readily extended to solar thermoelectric generators as well. In this case, the key element is a thin film heat absorber which collects the solar radiation concentrated through a suitable lens. In order to optimize the device efficiency one requires a wavelength-selective surface absorptance for this film to precisely match the solar spectrum distribution in the near UV, visible and near IR domains [32]. Whereas most experimental substrates considered up to now rely on specific materials, rather than engineered photonic crystals [33], the results reviewed in the previous sections clearly suggest that one may expect significant improvement in the spectral response of absorbing films based on aperiodic designs.

## 5. Conclusions

In this work we have reviewed the convenience of exploiting different aperiodic thin film designs for their use as coating materials in photonic devices requiring a high thermal emission spectrum combined with full reflectance properties at certain relatively broadband spectral windows. These aperiodic designs can be classified according to their increasing order of structural complexity. Thus, one can consider graded structures whose layer widths are determined by: (1) arithmetic or geometric series; (2) fractal self-similar patterns; (3) quasiperiodic sequences given by substitution rules; and (4) analytical functions able to encompass some of the previous refraction index profiles in a unified way. An important aspect supporting the use of these aperiodic order based designs concerns the purported robustness of their frequency spectra upon the unavoidable layer width fluctuations appearing during the growth of actual multilayers [7,28]. Accordingly, a deeper study of some of the proposed aperiodic designs may well deserve a closer experimental scrutiny. In doing so, it would also be interesting to consider coatings based on organic multilayers, as a suitable alternative to the inorganic materials (silicon, oxides, fluorides) mostly considered up to now [34,35,36].
